# Associations of Plastic Bottle Exposure with Infant Fecal Microbiota, Short-Chain Fatty Acids, and Growth

**DOI:** 10.21203/rs.3.rs-2454597/v1

**Published:** 2023-01-17

**Authors:** Heather Jianbo Zhao, Curtis Tilves, Moira Differding, Mingyu Zhang, Tiange Liu, Sara Benjamin-Neelon, Cathrine Hoyo, Truls Ostbye, Noel Mueller

**Affiliations:** Johns Hopkins Bloomberg School of Public Health; Johns Hopkins University Bloomberg School of Public Health; Johns Hopkins Bloomberg School of Public Health; Harvard Medical School; Johns Hopkins Bloomberg School of Public Health; Johns Hopkins University; Duke University Health System; Duke University Health System; Johns Hopkins Bloomberg School of Public Health

## Abstract

**Background:**

Plastic exposures have been shown to impact the microbiome, metabolism and growth of animals. However, no human studies have examined how plastic exposures are associated with fecal microbiota, microbial metabolites, or growth. Here we examine the association of plastic bottle feeding with infant fecal microbiota, microbial short-chain fatty acid (SCFA) metabolites, and anthropometry in the first year of life.

**Methods:**

462 infants from the prospective Nurture Birth Cohort were included to examine frequency of plastic bottle feeding (every feeding vs. less than every feeding) at 3 months with anthropometric outcomes (skinfolds, length-for-age, and weight-for-length) at 1 year. A subset of 64 and 67 infants were included in analyses examining the fecal microbiota and fecal SCFAs, respectively. Microbial taxa were measured by 16S rRNA gene sequencing of the V4 region and SCFA concentrations were quantified using gas chromatography at 3 and 12 months of age.

**Results:**

After adjustment for potential confounders, less frequent plastic bottle use was associated with lower fecal microbiota alpha Shannon diversity at 3 months (mean difference for plastic bottle used less than every feeding vs. every feeding = −0.53, 95% CI: −0.90, −0.17, p < 0.01) and lower propionic acid concentration at 3 months (mean log + 1 difference for plastic bottle used every feeding vs. less than every feeding = −0.53, 95% CI: −1.00, −0.06, p = 0.03). Furthermore, compared to infants who used plastic bottle at every feeding, infants who were plastic bottle-fed less frequently (1 −3 times/day) at 3 months had significantly lower length-for-age z-scores at 12 months (mean difference= −0.40, 95% CI: −0.72, −0.07, p = 0.016).

**Conclusion:**

Plastic bottle exposure may impact early infant gut microbiota and microbial SCFAs, which may in turn affect growth.

## Introduction

In the last decade, exposure to plastics become a growing public health concern (1), especially during vulnerable periods of development, such as infancy. Recent data suggest that infants have significantly higher fecal concentrations of plastic materials, with an estimated daily intake of 83 000 ng/kg body weight in infants compared to 5 800 ng/kg body weight in adults(2). The disproportionately high plastic exposure among infants is thought to be related to plastic-based products during feeding, such as bottle-feeding and sippy cups (2, 3). Although exposure to plastics has been previously linked with several adverse health outcomes in humans such as inflammatory bowel disease and respiratory illnesses (4–7), there is still limited data on how plastics affect metabolic health outcomes like obesity. Furthermore, there is limited data on potential mechanisms by which plastic exposure might alter metabolic health outcomes.

The human gut microbiome is the ensemble of microorganisms living in the intestinal tract and is largely determined by environmental factors, rather than genetics (8). Examination of the gut microbiome provides an opportunity to examine whether the provenance health effects of environmental factors, like plastic, start in the intestine. The human microbiome is purported to affect health outcomes by producing metabolites. In particular, the microbial production of short-chain fatty acid (SCFA) metabolites are thought to mediate microbiome-health outcome associations. SCFAs are products of the colonic microbiota from fibre and resistant starch. Specific SCFAs, such as acetate, propionate, and butyrate, play a key role in influencing host metabolism (9). To our knowledge, no studies have examined the association of exposure to plastics, particularly during infancy, with the gut microbiome or microbial metabolites (10). Additionally, fetal and childhood exposure to some plastics has been associated with interrupted development, such as lower infant birth weight, and higher BMI and waist circumference (11–13). However, the direct relation between infant plastic exposure and anthropometric growth variables in the first year of life has not been reported.

The primary aim of this study is to examine plastic bottle exposure frequency, measured by exposure to plastic bottle feeding, with the infant gut microbiota composition and diversity at 3 and 12 months of age in a longitudinal birth cohort study. We hypothesize that plastic bottle use is associated with gut microbiome diversity and composition. The secondary objectives of this study are to examine the relation between plastic exposure frequency and SCFA concentrations in the stool, and to examine the relation between plastic exposure frequency and anthropometric growth variables in the first year of life.

## Methods

### Study population

We used data from the prospective Nurture birth cohort (14) from central North Carolina. From 2013 to 2015, we recruited 666 women with a singleton pregnancy at 20–36 weeks of gestation from a county health department or private prenatal clinic. The cohort required mothers to be at least 18 years of age, have a singleton pregnancy, live at their current address a minimum of one year after delivery, and be able to speak and read English. We excluded mother-infant pairs if the infant was delivered prior to 28 weeks of gestation, had congenital abnormalities, or required 3 or more weeks of hospitalization postnatally. We received maternal written informed consent at recruitment and reconfirmed via phone shortly after delivery. Data collectors conducted home visits when infants were approximately 3, 6, 9, and 12 months of age, supplemented by monthly automated interactive voice response (IVR) calls in between visits. We asked a subsample of mothers to collect stool samples for microbiome analysis from infants at 3 and 12 months of age. A total of 64 infants had microbiome data, 67 had SCFA data, and 462 had anthropometric data. This study followed the guidelines of the Declaration of Helsinki and procedures involving human subjects and was approved by the Duke University Medical Center Institutional Review Board (human subjects committee) (PRO0036342). Although not a clinical trial, the study is registered at clinicaltrials.gov (NCT01788644).

### Primary Exposure - plastic bottle frequency use

Mothers reported the daily frequency of plastic bottle feeding among their infants every month up until 12 months during IVR calls. We looked at plastic bottle feeding at 3 months due to limited reporting beyond this time. We grouped infant’s plastic bottle use in six categories: <1 time a day, 1 time a day, 2–3 times a day, 4–5 times a day, 5 + times a day but not at every feeding, or at every feeding. In the analysis of the growth trajectories, we classified plastic bottle use into four groups: <1 time/day, 1–3 times/day, 4 + times/day, and every feeding. In the analysis for microbiota and SCFA, because of our limited sample size of 64 and 67, we classified plastic bottle use into two groups: less than every feeding and every feeding. Less than every feeding plastic bottle use included infants who used plastic bottle 0–5 times a day, and every feeding plastic bottle use included infants who used plastic bottle at every feeding.

### Covariate measurement

We abstracted data on delivery mode (C-section vs. vaginal delivery, with additional information on type of C-section), birth weight (kg), infant sex, and gestational age at birth (weeks) from medical records. We collected maternal age (year), ethnicity and race, number of people per household, pre-pregnancy weight (kg), height (meters), highest education obtained (high school or below, some college or above), household income (<$20 000 per year. vs. ≥$20 000 per year), and smoking status at the time of delivery (yes vs. no). We used self-reported maternal pre-pregnancy weight and height to calculate the pre-pregnancy body mass index (BMI measured in kg/m^2^) and categorized those with BMI 25 to 29.9 as overweight and BMI ≥ 30 as obese.

### Stool sample collection, microbial 16S rRNA gene extraction, and fecal SCFA measurement

We collected stool at 3- and 12-month home visits from diapers and transferred the stool to a 2 ml cryogenic vial (ThermoFisher). Subsequently, we froze the vial at − 80°C for later processing. The specimens were then thawed, and their DNA was extracted using the QIAgen MagAttract PowerSoil for KingFisher. We placed 0.5 g of stool in each bead plate well of the PowerSoil kit and extracted DNA per the manufacturer’s instructions. Then, we quantified DNA with the Quant-iT dsDNA high sensitivity kit (ThermoFisher). We measured fecal SCFA concentrations using gas chromatography (Thermo Trace 1310) paired to a flame ionization detector (Thermo). More specific methods regarding microbiome sequencing, data processing, and SCFA measurements are described elsewhere (15, 16).

### Growth variables

We measured infant weight, height, and skinfold thickness at 3, 6, 9, and 12 months. Trained data collectors measured infant weight in light clothing without shoes via a ShorrBoard Portable Length Board to nearest 1/8 pound; we measured infant height using a Seca Infant Scale to the nearest 0.1 inch. We rounded infant abdomen, subscapular, and triceps skinfold thicknesses measurements to the nearest millimetre using standard techniques (17). We repeated all measurements three times to reduce measurement error, with the final measurement recorded as the average three values. We calculated the age- and sex-specific weight-for-length z-score, the BMI-for-age z-score, and length-for-age z-score using the World Health Organization Child Growth Standards (18). We summed subscapular and triceps skinfolds thickness measures as a proxy for overall fatness.

### Data Analysis

#### Model adjustments and statistical significance thresholds

In all regression models, we considered associations before and after adjustment for potential confounding factors. We defined a confounder as a variable that has been previously associated with both our exposure (plastic bottle use) and our outcomes (gut microbiome, SCFAs, or growth parameters), but were not on the causal pathway. In our final multivariable model, we controlled for birth weight (kg), gestational age (weeks), maternal age (years), and household income (<$20,000 vs. >= $20,000). We excluded participants with missing covariates in the analyses.

#### Alpha diversity

We conducted linear regression analyses to estimate the association of plastic bottle frequency at 3 months with Shannon diversity at 3 months, with Shannon diversity at 12 months, and with the change in Shannon diversity from 3 months to 12 months.

#### Beta diversity analysis

We used the *R* package *phyloseq* (19)to estimate weighted UniFrac distances, a measure of pairwise community composition, and performed a Principal Coordinate Analysis (PCoA) to graphically assess clustering by variables of interest (20). We further employed permutational multivariate analysis of variance (PERMANOVA) from the *R* package *vegan* (21)with 9999 permutations to test for differences in weighted UniFrac distances before and after adjusting for covariates (21).

#### Microbial community differential abundance analysis

We used beta-binomial regression models that accounted for within-sample taxa correlation and variable sequencing depth from the R package *corncob* to test for differential abundance of taxa in infants at 3 and 12 months (22). We removed ASVs that were singletons or did not have a mean count at or above the 25th percentile in at least 10% of samples to avoid testing rare ASVs and improve power with our small sample population. We then used R package LOCOM to verify the taxa results from *corncob* (23).

#### SCFA regression models and correlations

We used univariate and multivariable linear regressions to examine the association of plastic bottle use frequency at 3 months of age and SCFA concentrations at 3 and 12 months of age and, including separate models for butyric acid, propionic acid, acetic acid, and total SCFAs (sum of butyric acid, propionic acid, acetic acid, isobutyric acid, valeric acid, and isovaleric acid concentrations). It is important to note that butyric acid, propionic acid, and acetic acid are functionally the same as butyrate, propionate, and acetate. We performed a natural log + 1 transformation on butyrate and proportionate concentrations at 3 and 12 months to normalize the distribution.

#### Growth analyses

We used univariate and multivariable generalized linear regressions to examine the association of plastic bottle use frequency at 3 months and growth outcomes at 12 months of age, including separate models for subscapular skinfolds, triceps skinfolds, abdominal skinfolds, subscapular + triceps skinfolds, and weight-for-length z-score. We then conducted a linear mixed model and growth trajectory for weight-for-length z-score at 3 months plastic bottle exposure, and a sensitivity trajectory analysis for 3 month breast feeding and plastic bottle exposure.

#### Sensitivity Analysis

We examined whether breastfeeding status modified the association of plastic bottle use at 3 months only with Shannon diversity, SCFA, and anthropometric outcomes by repeating the above analyses after stratifying by ever breastfeeding (vs. never) at 3 months. For example, in Shannon diversity there is 4 groups: never breastfed and every feeding plastic bottle use (reference group), never breastfed and less than every feeding plastic bottle use, ever breastfed and every feeding plastic bottle use, and ever breastfed and less than every feeding plastic bottle use).

We conducted sensitivity analyses to determine the robustness of our analyses for Shannon diversity, SCFA, and anthropometric outcomes, respectively. We repeated the above analyses in infants whose mothers were not smokers; who did not consume antibiotics during pregnancy; or who had higher educational status (i.e., had post-secondary education). We conducted further analysis for threats to confounding among infants who were plastic bottle-fed formula compared to those who were plastic bottle-fed breastmilk. We further examined our associations by other categories of bottle use frequency and formula feeding status (breastmilk vs. formula).

We considered a p-value of 0.05 as significant for analyses of alpha diversity, beta diversity, short-chain fatty acids, and anthropometrics. For differential abundances analyses, we considered a False Discovery Rate (FDR)-corrected threshold of 0.05.

## Results

A total of 462 infants had plastic bottle frequency data at 3 months and were included in the growth trajectory analysis. A total of 70 infants provided stool at either time point, but 3 had missing data on plastic bottle use frequency, leaving 67 infants in the analytic sample for microbiome as an outcome. A total of 64 infants had microbiota data at 3 months, 49 at 12 months, and 46 had microbiota data at both time points. A total of 67 infants had SCFA data at 3 months, 48 at 12 months, and 47 had SCFA data at both time points.

### Participant characteristics

Of the 462 infants included in the study, 301 (65.2%) were Black or African American race, 312 (67.5%) were from lower-income households (<$20,000 per year), and 269 (64.0%) had mothers with pre-pregnancy BMI ≥ 25kg/m^2^. At 3 months, 316 (68.4%) of infants were plastic bottle-fed at every feeding. A total of 231 (50.0%) of infants were ever breastfed at 3 months [Table 1].

### Gut microbiota alpha diversity

We summarized the estimated mean differences (β) in microbial Shannon diversity by plastic bottle use in Table 2. Infants who used plastic bottles less frequently (i.e., less than every feeding) at 3 months had a significantly lower Shannon diversity at 3 months of age (adjusted β = −0.53, 95% CI: −0.90, −0.17; unadjusted β = −0.46, 95% CI: −0.72, −0.20) compared to infants who used plastic bottles every feeding. Frequency of bottle use at 3 months was not significantly associated with Shannon diversity at 12 months of age (adjusted β = −0.19, 95% CI: −0.68, 0.29) [Supplemental Fig. 1], and was not associated with change in diversity between 3 and 12 months of age (adjusted β = 0.40, 95% CI: −0.12, 0.92). Additionally, across subgroups (i.e., mothers who were non-smokers, mothers who did not take antibiotics during pregnancy, mothers with high education attainment), Shannon diversity at 3 months continued to be lower among those who used plastic bottles less frequently compared to those with plastic bottle use in every feeding at 3 months [Supplemental Table 2].

We presented the estimated mean differences in microbial Shannon diversity by plastic bottle use and breast-feeding status at 3 months in Table 3. Infants who were ever breastfed at 3 months and used plastic bottles less frequently at 3 months had a significantly lower change in Shannon diversity at 3 months of age (adjusted β = −0.64, 95% CI: −1.03, −0.24; unadjusted β = −0.53, 95% CI: −0.82, −0.25) compared to infants who were never breastfed and had every feeding plastic bottle use. There were no significant differences between the other two categories compared to the reference group (i.e., never breastfed and every feeding plastic bottle use). The 4 groups were not associated with Shannon diversity at 12 months or change in Shannon diversity from 3 to 12 months.

### Gut microbiota beta diversity

Plastic bottle use frequency at 3 months was not significantly associated with Weighted UniFrac at 3 months (PERMANOVA p = 0.24, R^2^ = 0.019) or 12 months of age (PERMANOVA p = 0.78, and R^2^ = 0.012) [Supplementary Figs. 2a and 2b].

### Gut microbiota composition

**Figure 1:**
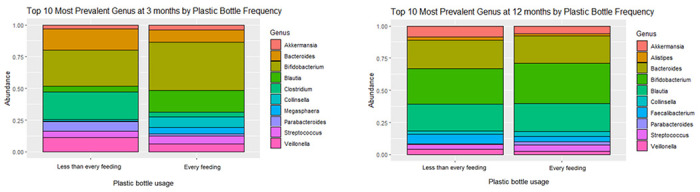
Unadjusted mean percent of a major bacterial genus at 3 and 12 months of age, by plastic bottle use.

The mean relative abundances of the top 10 bacterial genera at 3 and 12 months by plastic bottle use are shown in Fig. 1. The taxa associated at 12 months were similar to 3 months by plastic bottle exposure. Less frequent plastic bottle use was associated with differential abundance of 30 bacterial ASVs in the infant gut at 3 months and 26 bacterial ASV in the infant gut at 12 months of age, after adjustment for potential confounders [Supplemental Fig. 3].

### SCFA concentrations

Table 4 shows the estimated mean differences in SCFA concentrations (acetate, propionate, butyrate, and total SCFAs) at 3 months, 12 months, and change from 3 to 12 months by plastic bottle use at 3 months. Infants with less than every feeding plastic bottle at 3 months had a significantly lower propionic acid concentration at 3 months of age (adjusted mean log + 1 difference= −0.53, 95% CI: −1.00, −0.06; unadjusted mean log + 1 difference= −0.36, 95% CI: −0.70, 0.03) compared to infants with every feeding. Infants with less than every feeding plastic bottle at 3 months had a lower butyric acid concentration at 3 months of age (unadjusted mean log + 1 difference= −0.55 95% CI: −1.06, −0.06) but this association was somewhat attenuated after adjusting for confounders (mean log + 1 difference= −0.54, 95% CI: −1.25, 0.16). Significant differences were not observed in the 12 months analysis nor the change from 3 to 12 months. Acetic acid, butyric acid, and total SCFAs mean differences were not significant at 3 months, 12 months, or the change from 3 to 12 months.

### Anthropometric growth variables

**Figure 2:**
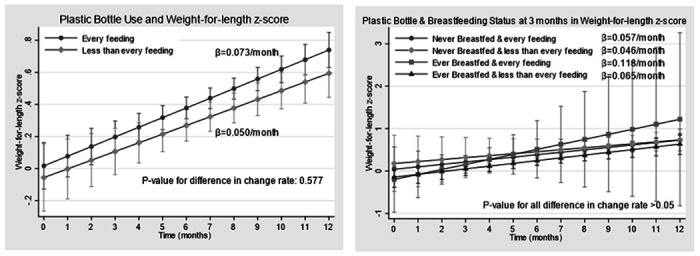
**a.** Weight-for-length z-score trajectory in infants at 3 months of age who were plastic bottle fed every feeding and less than every feeding. Controlled for birth weight (kg), gestational age (weeks), maternal age (years), household income (<$20 000 per year vs. ≥$20 000 per year). **b.** Weight-for-length z-score trajectory in infants at 3 months of age who were plastic bottle fed in everyday feeding and less than everyday feeding and at 3 months of age who were ever or never breastfed. Controlled for birth weight (kg), gestational age (weeks), maternal age (years), household income (<$20 000 per year vs. ≥$20 000 per year).

Table 5 shows the anthropometric growth variables measured at 12 months (subscapular skinfolds, triceps skinfolds, abdominal skinfolds, subscapular + triceps skinfolds, BMI z-score, infant length-for-age z-score, and weight-for-length z-score). Infants who were plastic bottle-fed 1–3 times/day at 3 months had a significantly lower length-for-age z-score at 12 months (adjusted β = −0.40, 95% CI: −0.72, −0.07; unadjusted β = −0.38, 95% CI: −0.69, −0.06) compared to infants who used a plastic bottle everyday. There were no significant mean differences in weight-for-length z-score across different plastic bottle use categories in the trajectory analysis [Figure 2].

## Discussion

In our prospective birth cohort of racially diverse mother-infant dyads from North Carolina, we found that less frequent plastic bottle use (i.e., using plastic bottles less than every feeding vs. every feeding) was associated with lower fecal microbiota diversity at 3 months of age. Less frequent plastic bottle use was also associated with a lower fecal propionic acid concentration at 3 months of age. Furthermore, infants who were plastic bottle-fed 1–3 times per day (vs. every feeding) had a significantly lower length-for-age z-score at 12 months. However, other plastic bottle feeding categories (4+/day and < 1/day) were not associated with linear growth, nor were they associated with weight.

We are not aware of other studies in humans that have examined the effect of plastic bottle use on infant gut microbiota composition, microbiota diversity, and SCFAs. However, in an animal study, introducing polystyrene microplastics significantly altered the alpha diversity of mice gut microbiota (5). The investigators of this study also reported that the abundance of the genera *Blautia, Bifidobacterium, Prevotella,* and *Parabacteroides* decreased as plastic exposure increased, as contrary to our 3-month analysis which showed that the abundance of these three genra increased as plastic exposure increased (Supplementary Material). Differences between our studies may be due to several factors, first being that we studied humans and Jin et al. studied mice, we studied plastic bottles and they studied microplastics.

Our finding that plastic bottle use was associated with lower fecal propionic acid concentration is of particular interest. Propionic acid is hypothesized to lower lipogenesis in tissues (24). A study in mice found that administering propionic acid was protective against diet-induced obesity, insulin resistance, and reduced food intake (25). Furthermore, it is possible that having less propionic acid detected in the feces could indicate that more propionic acid is being absorbed in the gut and translocated to circulation. Another notable SCFA finding in our study was the association of plastic bottle use with butyric acid, which is a major energy source for colonocytes and a regulator of gene expression in glucose metabolism (9, 26–28). A study in infants found that Shannon diversity at 3 months of age was positively correlated with fecal butyric acid (15). However, our study found that infants who used plastic bottle at every feeding had a higher butyric acid concentration, albeit not significant, and a lower alpha diversity. This discrepancy could be because higher butyric acid in the stool may indicate that metabolites are being excreted instead of absorbed (29).

It is important to study plastics in infants because microplastic levels have been found to be higher in infants compared to adults, likely due to infants’ unique dietary exposures (2, 3). For example, infants have extensive use of plastic products such as bottles and sippy cups. Infant formula prepared in plastic bottles can release millions of microplastics (3), potentially exacerbating the gut microbiota. The Canadian Healthy Infant Longitudinal Development (CHILD) birth cohort found that formula-fed infants had a greater diversity at 3 months of age (30). This is further reflected in our results in which formula-fed infants with less than every feeding plastic bottle had a higher Shannon diversity than those who were breast-fed. Furthermore, a Norwegian birth cohort found that higher diversity at 3 months may be associated with an increased risk of overweight in adulthood (31). Thus, plastic bottle exposure may contribute to the risk of obesity later in life, but not so much to infant adiposity as observed in this study.

### Strengths and Limitations

Our study is strengthened by its longitudinal design, active follow-up, the detailed infant growth data and covariate data, high racial diversity and low socioeconomic status, and the joint measurement of the fecal microbiome and metabolome. There were also several limitations to our study. First, we relied on 16S rRNA sequencing data, which may not always allow for species level resolution. Secondly, we did not have blood measurements available to measure circulating SCFAs, which differ in prevalence in the stool compared to blood. Third, plastic exposure was not measured objectively (i.e., using a biomarker). Fourth, we used a small sample from one region in North Carolina, which may limit generalizability. Fifth, even though mothers may have plastic bottle-fed their infants, the substance mothers put in the plastic bottle, whether it be breastmilk or formula, may differ. Nevertheless, we addressed this limitation by conducting a subgroup analysis for joint effects in plastic bottle use frequency and status of formula feeding (breastmilk vs. formula) [Supplementary Material]. Another limitation is the potential for reporting bias. Mothers may have underreported plastic bottle feeding if they believed that direct breastfeeding is a better practice. Furthermore, we cannot identify the type of plastic materials used in the bottle, and whether different materials could lead to different concentrations of plastics released in the formula. Lastly, despite covariate adjustment, we cannot rule out the possibility of unmeasured or residual confounding.

## Conclusion

At 3 months, infants who were plastic bottle-fed during less than every feeding had a lower alpha diversity than infants who were plastic bottle-fed in every feeding. Encouraging mothers to lower their use of plastic bottles, limiting microwaves to heat plastic bottles that could release more plastic particles into the formula, or replacing plastic with glass bottles may be beneficial to their infant’s gut microbiota. Nevertheless, larger longitudinal gut microbiota studies, with more specific analysis of the concentrations and types of plastic particles, are required to confirm whether the microbiome is impacted by plastic exposure in the first few months of life, and whether the infant microbiome is causal to developing health conditions such as greater weight gain or reduced linear growth, which we did not identify in our cohort.

## Figures and Tables

**Table 1:** Characteristics of mother-infant pairs in the Nurture birth cohort study by plastic bottle use frequency at 3 months of age.

	Every feeding N = 316	4+ /day N = 43	1–3/day N = 60	< 1/day N = 43
**C-section**	117 (37%)	16 (37%)	20 (33%)	6 (14%)
**Pre-pregnancy BMI ≥ 25kg/m2**	215 (68%)	27 (63%)	36 (60%)	18 (43%)
**Maternal age (SD)**	26.59 (5/40)	28.84 (5.65)	29.81 (6.50)	39.28 (5.64)
**Infant male sex**	161 (50.95%)	21 (48.84%)	28 (47.67%)	23 (54.76%)
**Infant Black race**	232 (73.42%)	25 (58.14%)	33 (55.00%)	11 (26.19%)
**Ever breastfed at 3 months**	95 (30.06%)	36 (87.72%)	58 (96.67%)	42 (100.00%)
**Maternal low educational achievement**	175 (55.38%)	18 (41.86%)	11 (18.33%)	8 (19.05%)
**Household income <$20,000**	241 (77.24%)	26 (60.47%)	31 (52.54%)	14 (33.33%)
**Maternal antibiotics during pregnancy**	100 (31.65%)	15 (34.88%)	11 (18.33%)	18 (42.86%)
**Current smoker**	45 (14.24%)	3 (6.98%)	2 (3.33%)	0 (0.00%)
**Infant birth weight, kg (SD)**	3.20 (0.53)	3.20 (0.41)	3.37 (0.49)	3.26 (0.53)
**Infant gestational age in weeks (SD)**	38.59 (1.51)	38.81 (1.75)	38.93 (1.41)	38.71 (1.38)
**Number of people in household (SD)**	2.50 (1.41)	2.09 (1.17)	2.10 (1.28)	2.38 (1.41)

**Table 2:** Unadjusted and multivariable-adjusted mean difference (and 95% Confidence Interval) in fecal microbiota alpha diversity (Shannon Diversity) assessed at 3 and 12 months of age according to plastic bottle use at 3 months of age.

	Difference in Shannon diversity at 3 months (n = 64)		Difference in Shannon diversity at 12 months (n = 46)		Difference in the change in Shannon diversity from 3 months to 12 months) (n = 46)	
	Crude	Adjusted	Crude	Adjusted	Crude	Adjusted
**Plastic bottle provided every feeding**	0.0 (ref) n = 49	0.0 (ref) n = 49	0.0 (ref) n = 34	0.0 (ref) n = 34	0.0 (ref) n = 34	0.0 (ref) n = 34
**Plastic bottle provided less than every feeding**	−0.46 *** (−0.72, −0.20) n = 15	−0.53 ** (−0.90, −0.17) n = 15	−0.07 (−0.44, 0.29) n = 12	−0.19 (−0.68, 0.29) n = 12	0.37 (−0.01, 0.76) n = 12	0.40 (−0.12, 0.92) n = 12

Multivariable-adjusted models included adjustment for birth weight (kg), gestational age (weeks), maternal age (years), household income (<$20 000 per year. vs. ≥$20 000 per year)

* = p < 0.05,

** = p < 0.01,

*** = p < 0.001

**Table 3:** Unadjusted and multivariable-adjusted mean difference (and 95% Confidence Interval) in fecal microbiota alpha diversity (Shannon Index) assessed at 3 and 12 months of age according to plastic bottle use at 3 months and breastfeeding status at 3 months.

	Difference in fecal microbiota alpha diversity Shannon Index at 3 months (n = 64)		Difference in fecal microbiota alpha diversity Shannon Index at 12 months (n = 46)		Difference in the change in fecal microbiota alpha diversity Shannon Index from 3 months to 12 months) (n = 46)	
	Crude	Adjusted	Crude	Adjusted	Crude	Adjusted
**Never breast fed at 3 months and used plastic bottle every feeding**	0.0 (ref) n = 36	0.0 (ref) n = 36	0.0 (ref) n = 28	0.0 (ref) n = 28	0.0 (ref) n = 28	0.0 (ref) n = 28
**Never breastfed at 3 months and used plastic bottle less than every feeding**	−0.06 (−0.58, 0.46) n = 3	−0.19 (−0.77, 0.29) n = 3	0.24 (−0.89, 1.37) n = 1	0.11 (−1.05, 1.27) n = 1	0.19 (−1.00, 1.37) n = 1	0.05 (−1.20, 1.30) n = 1
**Ever breastfed at 3 months and used plastic bottle every feeding**	0.09 (−0.19, 0.36) n = 13	0.18 (−0.13, 0.49) n = 13	0.15 (−0.35, 0.65) n = 6	0.20 (−0.34, 0.73) n = 6	−0.07 (−0.59, 0.46) n = 6	−0.17 (−0.75, 0.40) n = 6
**Ever breastfed at 3 months and used plastic bottle less than every feeding**	−0.53 *** (−0.82, −0.25) n = 12	−0.64 ** (−1.03, −0.24) n = 12	−0.07 (−0.47, 0.32) n = 11	−0.25 (−0.80, 0.30) n = 11	0.38 (−0.04, 0.79) n = 11	0.46 (−0.13, 1.05) n = 11

Multivariable-adjusted models included adjustment for birth weight (kg), gestational age (weeks), maternal age (years), household income (<$20 000 per year. vs. ≥$20 000 per year)

* = p < 0.05,

** = p < 0.01,

*** = p < 0.001

**Table 4:** Unadjusted and multivariable-adjusted mean difference (and 95% Confidence Interval) in fecal short-chain fatty acid concentrations (μmol/g) assessed at 3 and 12 months of age according to plastic bottle use at 3 months of age.

	Acetic Acid Mean difference		Propionic Acid±Mean difference		Butyric Acid±Mean difference		Total SCFAs Mean difference	
	Crude	Adjusted	Crude	Adjusted	Crude	Adjusted	Crude	Adjusted
SCFAs at 3 months								
**Plastic bottle provided every feeding**	0.0 (ref) n = 52	0.0 (ref) n = 52	0.0 (ref) n = 52	0.0 (ref) n = 52	0.0 (ref) n = 51	0.0 (ref) n = 51	0.0 (ref) n = 52	0.0 (ref) n = 52
**Plastic bottle provided less than every feeding**	1.90 (−23.90, 16.45) n = 15	1.90 (−26.00, 29.79) n = 15	−0.36* (−0.70, −0.03) n = 15	−0.53* (−1.00, −0.06) n = 15	−0.55* (−1.06, −0.03) n = 14	−0.54 (−1.25, 0.16) n = 14	−15.22 (−42.50, 12.05) n = 15	−13.51 (−51.18, 24.16) n = 15
SCFA at 12 months								
**Plastic bottle provided every feeding**	0.0 (ref) n = 36	0.0 (ref) n = 36	0.0 (ref) n = 36	0.0 (ref) n = 36	0.0 (ref) n = 36	0.0 (ref) n = 36	0.0 (ref) n = 36	0.0 (ref) n = 36
**Plastic bottle provided less than every feeding**	−17.12 (−39.68, 5.44) n = 12	−26.40 (−56.60, 3.80) n = 12	−0.12 (−0.48, 0.25) n = 12	−0.11 (−0.58, 0.35) n = 12	0.00 (−0.49, 0.49) n = 12	−0.08 (−0.74, 0.57) n = 12	−22.13 (−54.70, 10.45) n = 12	−32.72 (−75.44, 9.99) n = 12
Change from 3 to 12 months								
**Plastic bottle provided every feeding**	0.0 (ref) n = 35	0.0 (ref) n = 35	0.0 (ref) n = 33	0.0 (ref) n = 33	0.0 (ref) n = 34	0.0 (ref) n = 34	0.0 (ref) n = 35	0.0 (ref) n = 35
**Plastic bottle provided less than every feeding**	−19.33 (−51.56, 12.90) n = 12	−33.54 (−75.57, 8.49) n = 12	0.16 (−0.29, 0.60) n = 12	0.37 (−0.16, 0.89) n = 12	0.63 (−0.07, 1.34) n = 11	0.52 (−0.41, 1.46) n = 11	−14.02 (−58.97, 30.94) n = 12	−24.28 (−81.14, 32.58) n = 12

±Natural log + 1 transformation used for butyric acid and propionic acid at 3 and 12 months

Multivariable-adjusted linear models included adjustment for birth weight (kg), gestational age (weeks), maternal age (years), household income (<$20 000 per year. vs. ≥$20 000 per year)

* = p < 0.05

**Table 5:** Unadjusted and multivariable-adjusted mean difference (and 95% Confidence Interval) in growth parameters assessed at 12 months of age according to plastic bottle use at 3 months of age.

		Subscapular skinfolds	Abdominal skinfolds	Triceps skinfolds	Subscapular + Triceps skinfolds	BMI z- score	Length for age z- score	Weight for length z- score
**Crude**	**Plastic bottle provided every feeding**	n = 240 reference	n = 239 reference	n = 239 reference	n = 239 reference	n = 240 reference	n = 240 reference	n = 250 reference
	**Plastic bottle provided 4+/day**	n = 37 −0.40 (−0.89, 0.09)	n = 37 −0.12 (−0.82, 0.58)	n = 37 −0.15 (−0.83, 0.54)	n = 37 −0.53 (−1.58, 0.51)	n = 37 −0.34 (−0.70, 0.02)	n = 37 −0.08 (−0.29, 0.44)	n = 37 −0.32 (−0.68, 0.03)
	**Plastic bottle provided 1–3/day**	n = 55 −0.09 (−0.50, 0.33)	n = 54 0.04 (−0.56, 0.64)	n = 55 −0.04 (−0.63, 0.54)	n = 55 −0.12 (−1.01, 0.76)	n = 53 0.03 (−0.28, 0.33)	n = 53 −0.38 * (−0.69, −0.06)	n = 53 −0.03 (−0.33, 0.28)
	**Plastic bottle provided < 1/day**	n = 39 −0.29 (−0.77, 0.19)	n = 39 0.38 (−0.31, 1.06)	n = 39 0.24 (−0.43, 0.92)	n = 39 −0.03 (−1.05, 0.99)	n = 39 −0.15 (−0.49, 0.20)	n = 39 −0.19 (−0.55, 0.17)	n = 39 −0.17 (−0.52, 0.18)
**Adjusted**	**Plastic bottle provided every feeding**	n = 236 reference	n = 235 reference	n = 235 reference	n = 235 reference	n = 236 reference	n = 236 reference	n = 236 reference
	**Plastic bottle provided 4 + t/day**	n = 37 −0.31 (−0.81, 0.18)	n = 37 −0.01 (−0.71, 0.69)	n = 37 −0.17 (−0.87, 0.53)	n = 37 −0.48 (−1.54, 0.57)	n = 37 −0.31 (−0.66, 0.04)	n = 37 −0.14 (−0.23, 0.50)	n = 37 −0.28 (−0.62, 0.07)
	**Plastic bottle provided 1-3/day**	n = 54 −0.07 (−0.51, 0.36)	n = 53 0.08 (−0.54, 0.70)	n = 54 −0.13 (−0.74, 0.47)	n = 54 −0.20 (−1.13, 0.73)	n = 53 −0.04 (−0.35, 0.27)	n = 53 −0.40* (−0.72, −0.07)	n = 53 −0.10 (−0.40, 0.21) p = 0.50
	**Plastic bottle provided < 1/day**	n = 39 −0.19 (−0.69, 0.31)	n = 39 0.47 (−0.24, 1.19)	n = 39 0.12 (−0.59, 0.83)	n = 39 −0.06 (−1.13, 1.01)	n = 39 −0.13 (−0.49, 0.22)	n = 39 −0.16 (−0.09, 0.08)	n = 39 −0.14 (−0.50, 0.21)

Multivariable-adjusted linear models included adjustment for birth weight (kg), gestational age (weeks), maternal age (years), household income (<$20 000 per year vs. ≥ $20 000 per year).

* = p < 0.05

## Data Availability

The data that support the findings of this study are available from the Nurture birth cohort principal investigator. However, some restrictions apply to the availability of these data, which were used under license for the current study, thus are not publicly available. Data are however available from the authors upon reasonable request and with permission from Duke University Medical Center.
